# Effect of Potassium Ions on Protoplast Generation during Yeast Induction from *Mucor circinelloides* Tieghem

**DOI:** 10.5402/2013/734612

**Published:** 2012-11-11

**Authors:** C. O. Omoifo

**Affiliations:** Department of Crop Science, Ambrose Alli University, Ekpoma, Nigeria

## Abstract

*Mucor circinelloides* aerobically exhibits coenocytic thallic growth habit with straight and circinate sporangiophores which culminate in globose or pyriform columellae enclosed within sporangial walls. It undergoes dimorphic switch with its conversion to multipolar budding yeast-like cells or thallic conidia. This paper confirms the induction of plurality of reproductive structures of the pleomorphic microorganism in minimal medium. Furthermore, construction of pH differentials at inflection points in the biphasic profiles during sporangiospore-yeast transformation indicated the intensity of H^+^ release from intracellular medium of the growing microorganism in a study conducted with K^+^ levels (0.0, 0.5, 0.7, 0.9, 1.0,1.10 g/L)-mediated broths. Optimum proton release was at 0.00 and 1.0 g/L K^+^-supplemented broths, but specific growth rate was least in the latter. It also coincided with a preponderance of neoplastic units, protoplasts, and terminal budding yeast cells. On either side of this K^+^ level, variation in morphologies, including neoplasts, protoplasts, septate hyphae, thallic, holothallic, and holoblastic conidia, was greater, although olive-green septate hyphae with vesicular conidiogenous apparatus occurred at all K^+^ levels tested. This study suggested that following the establishment of transmembrane pH gradient across protoplast membrane, operation of Mitchellian proton pump was further promoted, thus leading to active transport mechanism, a prelude to yeast morphology induction.

## 1. Introduction

Fungi produce spores so as to reproduce the species. Zygomycetes are well known for zygospore formation in sexual reproduction but sporangiospores in asexual reproduction. *Mucor circinelloides *has large multispored sporangia, which contain well-defined columellae. Although monotypic with coenocytic thallic growth, it speciates by the possession of two types of columellae, which are spherical and pyriform (ending with a truncate base), and two types of sporangiophores, upright and circinate—from which it derives its name [1]. Sporangiophores are abundant and this gives the colony a compact appearance. This is the common expression in aerobic environment. 

*M. circinelloides *(syn: *M. racemosus*) exhibits dimorphism as it converts to multipolar budding yeast-like cells in CO_2_ atmosphere [2]. An additional growth form, thallic conidia formation, like the arthric conidia of the nature of *Geotrichum candidum* [3], was shown by McIntyre et al. [4] when this fungus was grown in minimal Vogels medium. The formation of thallic conidia by *M. circinelloides *was confirmed by Lübberhüsen et al. [5] who found that such conidia were multinucleate. However, McIntyre and his colleagues showed in their study that thallic conidia formation was as a result of extreme sensitivity to oxygen [4]. 

*M. circinelloides *is useful candidate for research as it has been shown to have high potential for application industrially, either in the production of enzymes or recombinant proteins. Thus, the need was elicited for an understanding of its physiological processes for evolution of specific morphologies, since form of growth is a process parameter in any industrial set-up [5]. In response to this advice, Omoifo and Awalemhen [6] studied the growth of this organism in well-defined minimal medium and found that the microorganism exhibited a plurality of structures. 

 During the transformation process new morphologies were formed upon preexisting ones; thus diverse reproductive structures were formed. Induced forms included transient forms, which differ markedly in shape and structure, evolved into the correct spatial relationships relative to one another, as well as persistent structures. So we have had for *M. circinelloides* induced asexual structures such as holoblastic, holothallic, and thallic conidia and yeast cells [6], each of which is proliferate [7]. It was shown in the study of Omoifo and Awalemhen [6] that during the growth process, intermedial ionic communication occurred and this appeared to correlate with the occurrence of specific morphologies. It was further shown that a sigmoid growth pattern was described in the 1.0 g/L K^+^–0.10 g/L Na^+^ level, and to a lesser extent in 1.0 g/L K^+^–0.20 g/L Na^+^ supplemented broth in which terminal budding yeast cells predominated.

 In this dimorphic switch, it is pertinent to ask how one form directionally organizes to yield the next transient form, or whether the directional assembly of morphogenetic sequences is spontaneous or controlled. In an attempt to throw some light on this phenomenon of dimorphic switch from the filamentous form, in this study we vary one elemental component of the medium we are developing for yeast induction, and using *M. circinelloides* as test organism we here show the effect of K^+^ on the process of yeast induction. We found that protoplasts were copiously generated at optimum K^+^ level, but H^+^ release intensity from intracellular medium appeared to have significant effect on the generation of protoplasts, which subsequently led to the nonpersistent yeast morphology.

## 2. Methods

### 2.1. Fungal Strain and Maintenance

The collection of samples of soil and decayed fruits of soursop, *Annona muricata *L., from which the organism, *M. circinelloides* Tieghem, was first isolated was reported in a previous study [7]. It has been used in other studies [8–11]. It was maintained as glucose-yeast extract-peptone (GYP : 10 : 03 : 5 g/L) solid cultures where it exhibited filamentous growth habit. A fresh culture was prepared after seven days.

### 2.2. Inoculum Preparation for Growth Studies

Inoculum was obtained by pouring sterile deionized distilled water over aerobic growth and a sterile glass rod gently passed over the surface so as to dislodge the spores. The suspension was poured into centrifuge tubes and spores washed by centrifuging at 5000 rpm for 7 min at 25°C in an MSE 18 centrifuge. The wash was decanted; sediment resuspended and further washed with two changes of sterile deionized distilled water. Spore count was taken with Neubauer haemocytometer (BSS No. 784, Hawksley, London vol. 1/4000) and was adjusted to 1 m^6^ spores per mL in sterile deionized distilled water, with the aid of a tally counter.

### 2.3. Reagents and Culture Media

All the reagents for the synthetic culture medium were obtained from BDH Laboratory supplies (Poole, UK). Media were prepared per litre of glucose, 10.0 g; (NH_4_)_2_SO_4_, 5.0 g; MgSO_4_·2H_2_O, 2.0 g; FeSO_4_·7H_2_O, 0.10 g; NaCl, 0.10 g; MnCl_2_, 0.065 g; CuSO_4_·7H_2_O, 0.06 g; ZnSO_4_·5H_2_O, 0.06 g. Media were prepared in 5000 mL beakers. Weights of buffer components 0.2 M Na_2_HPO_4_, 0.1 M citrate were obtained using H54AR Mettler balance and added to the beaker. Since the effect of K^+^ was to be tested, each of the duplicate flasks was incorporated with the various concentrations of KH_2_PO_4_: 0.0, 0.50, 0.70, 0.90, 1.00, and 1.10 g/L. The pH was adjusted to 4.5 with 2 N NaOH or 1 N HCl, using a Cole-Parmer pH Tester model 59000, in the 5000 mL beakers before dispensing into an 80 mL of broth in each of the duplicate 250 mL Erlenmeyer flasks for each test. The solution in each flask was made up to 100 mL with glass distilled deionized water and sterilized at 121°C for 15 min.

### 2.4. Inoculation, Growth Conditions, and Sample Collection

A 1 mL of spore suspension was drawn and inoculated into each broth flask using a 0.5 mL rubber suctioned pipette in a laminar flow chamber, model CRC, HB-60-180. The inoculum flask was shaken at each operation so as to keep the spores in suspension. Each culture flask was then shaken for 30 seconds and thereafter incubated at 20°C in a preset cooled Gallenkamp incubator. At 24 h interval the culture flasks were brought to the inoculating chamber. The flasks were shaken; 10 mL of broth was withdrawn with presterilized pipette, one for each culture medium, and deposited into factory-sterilized plastic sample tubes, prelabeled for each experiment. The culture flasks were returned for further incubation and samples kept at –18°C until analysis. Solid cultures were prepared in glucose-yeast extract-peptone agar (GYPA: 10.0–0.3–5.0–20 g/L) and incubated at 28°C, ambient.

### 2.5. Biomass Determination

Culture broth samples were thawed up to room temperature before biomass determination. This was done by measurement of optical density at 520 nm. This wavelength was chosen because ordinarily FeSO_4_ impacted greenish coloration on culture broth. Absorbance was determined using the Grating Spectrophotometer CE 303 (Cecil Instruments, Cambridge, UK). For pH determination, the culture suspensions were centrifuged at 5000 rpm for 7 min., 25°C (MSE 18). The clear media were each decanted and pH determined using pH meter 7020 (Electronic Industries Ltd). Microscopic examination was done after staining slide preparations with lactophenol-in-cotton blue. Micrographs were obtained with a Leitz Wetzlar Ortholux microscope (Germany) attached with an Ernst Leitz camera.

### 2.6. Analysis

This was done in Microsoft Excel environment. Growth data were log_10_ transformed. 

## 3. Results

### 3.1. Effect of K^+^ Concentration on H^+^ Release by *M. circinelloides*

**Figure 1:**
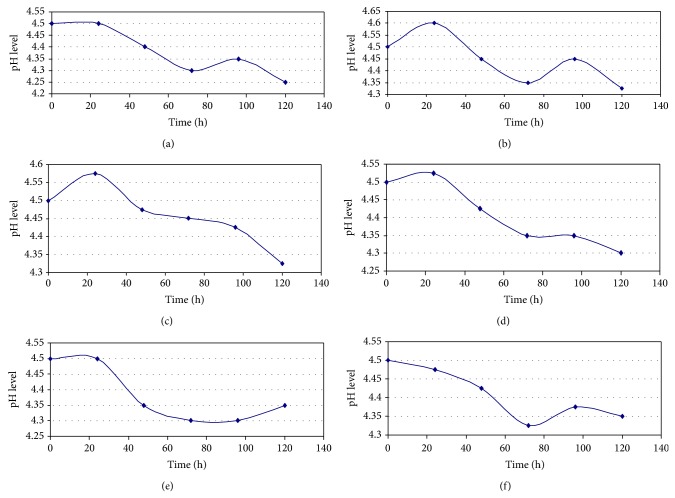
Deviation in pH-time course in the growth of *M. circinelloides* Tieghem incubated at different concentrations of K^+^ for 120 h at 20°C, ambient; (a), 0.0, (b), 0.50, (c), 0.70, (d), 0.90, (e), 1.0, (f), 1.10 g/L K^+^.

**Figure 2:**
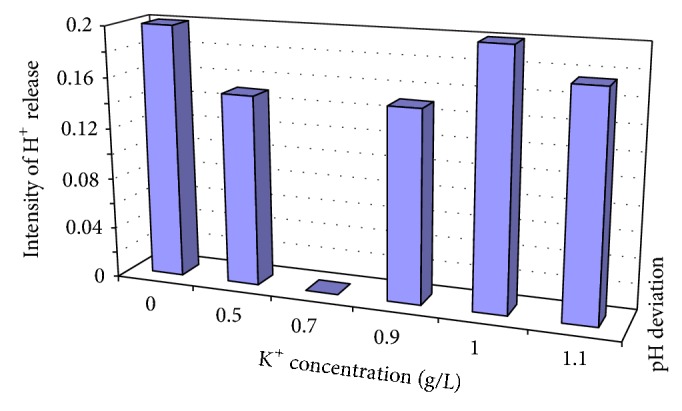
Intensity of H^+^ release after 72 h of growth from intracellular medium of *M. Circinelloides* Tieghem cultivated in K^+^-mediated glucose-substrate multi-ionic broth at pH 4.5, temp. 20°C, ambient.

The H^+^ release profile of *M*. *circinelloides *as affected by K^+^ conc. was shown in Figures 1(a)–1(f). At all levels of K^+^ tested, there was an initial tendency towards alkalinity. But the pH soon decreased below the initial value after 24 h, reaching a minimum level after 72 h of growth. Then the course reversed. The upward trend in recorded pH after inflexion was again broken as it reversed after 96 h and remained so till termination of the experiments. It was only at 1.0 g/L K^+^, where the 2nd phase point of inflexion did not occur, that the pH profile assumed a 2-phase construct (Figure 1(e)). The maximum pH differential occurred at this point of inflexion, that is, at 72 h after inoculation. Similar consideration yielded optimum values at inflexion points in pH profiles for the other K^+^ levels, where increase in acidity meant addition of H^+^ to the bulk medium. Such H^+^ were extruded from intracellular medium of the growing microorganism.  Figure 2 showed the intensity of H^+^ release from the intracellular medium at inflexion points, 72 h after inoculation, in relation to variation in K^+^ concentration. It was optimal at 0.00 and 1.00 g/L K^+^ but least at 0.70 g/L K^+^, although the medium became more acidic than the commencement pH level, at termination of experiments.

### 3.2. Effect of K^+^ Concentration on Growth of *M. circinelloides*

**Figure 3:**
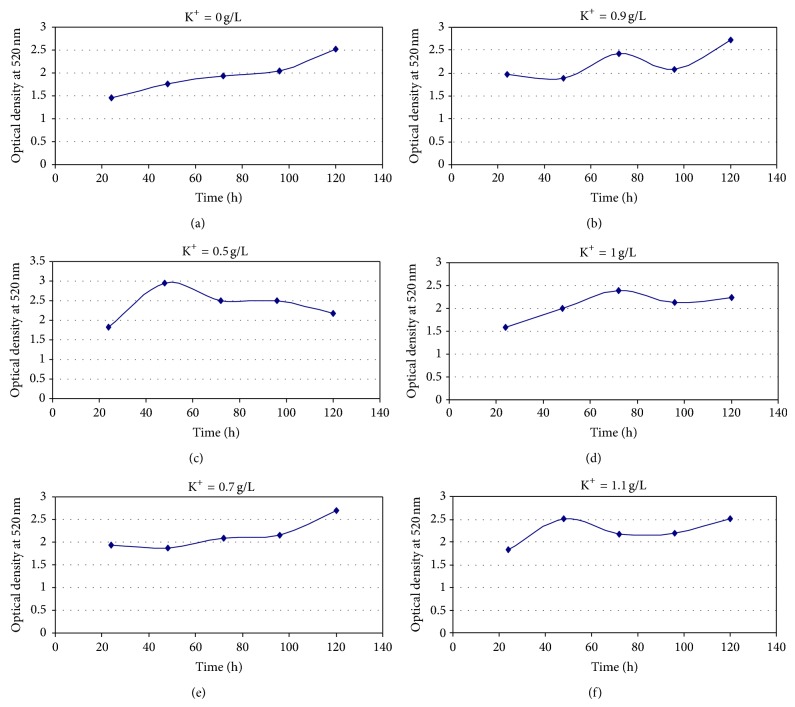
Growth profiles of *M. circinelloides *Tieghem cultivated in K^+^-mediated glucose-substrate multi-ionic broth for 120 h at pH4.5, temp. 20°C, ambient.

Growth profiles of *M*. *circinelloides*, as affected by variation in K^+^ concentrations, were shown in Figure 3. Time-course study in control experiments gave more or less steady rise in OD reading but sigmoid growth pattern was not described (Figure 3(a)). Variation occurred in the growth profiles at the different K^+^ levels, but quite like the control experiments, none assumed sigmoid growth pattern (Figures 3(b)–3(f)). The log transformed OD reading was maximal after 48 h, in the 0.5 g/L and 1.10 g/ L broths, and thereafter fell to a level bellow that, or at par with the control tests, which was, in turn, less than those determined for the 0.7 and 0.9 g/L  K^+^ broths. Final OD determination in the control tests was however higher than the final value obtained at 1.0 g/L K^+^.

**Figure 4:**
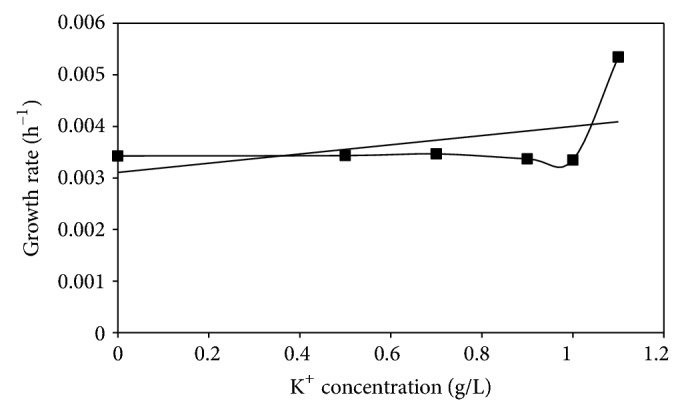
Specific growth rate of *Mucor circinelloides *Tieghe cultivated in buffered glucose-substrated multi-ionic broth at different levels of K^+^ for 120 h at pH4.5, temp. 20°C, ambient.

When a plot of the specific growth rates at the different K^+^ levels was obtained, it was found to be similar at K^+^ 0.00, 0.50, 0.70 g/L, decreased slightly at 0.9 g/L K^+^ but was least at 1.0 g/L  K^+^ and thereafter shot to a new high level at 1.10 g/L K^+^ (Figure 4).

### 3.3. Effect of K^+^ Concentration of Morphology of *M. circinelloides*

**Figure 5:**
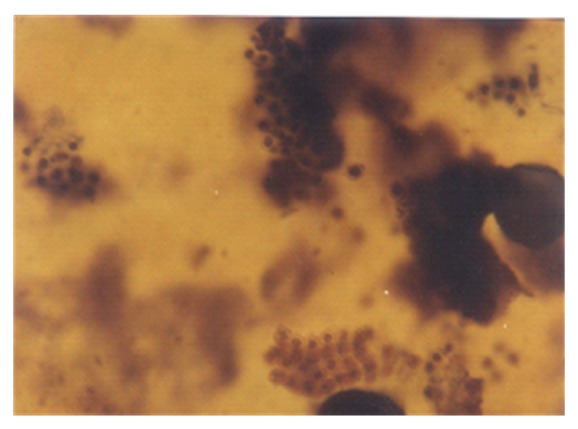
Vesicular conidial head group of *M. circinelloides *induced in glucose-substrated multi-ionic broth.

**Figure 6:**
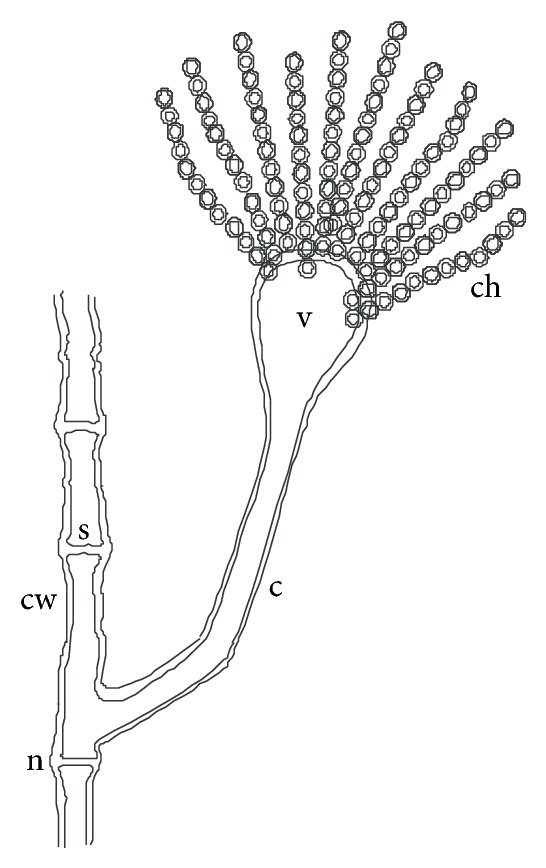
Diagram showing septate hypha, conidiophore, and vesicular concurrent catenate conidia of *M. circinelloides *coinduced with holoblastic, holothallic, and thallic conidia when cultivated in buffered glucose-substrated multiionic broth mediated with 0.00 g/L K^+^ for 120 h at pH 4.5, temp. 20°C, ambient; c: conidiophore, ch: catenate conidia, cw: cell wall, n: hyphal notch, s: septum, v: vesicle.

**Figure 7:**
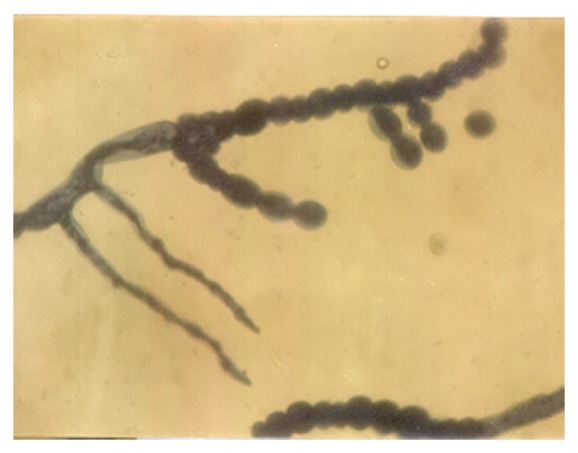
Thallic conidia of *M. circinelloides *Tieghem induced in glucose-substrated multiionic broth at ×400 mag.

**Figure 8:**
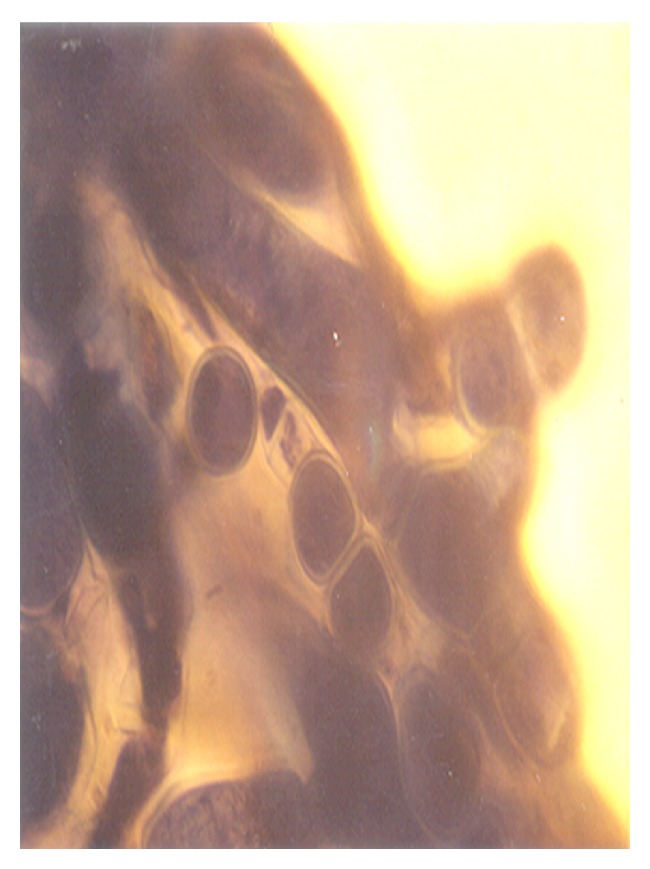
Thallic conidia of *M. circinelloides *Tieghem induced in glucose-substrate multiionic broth at ×1000 mag. These conidia, Figures 7 and 8, were mistakenly referred to as enterothallic conidia in a previous report [6].

**Figure 9:**
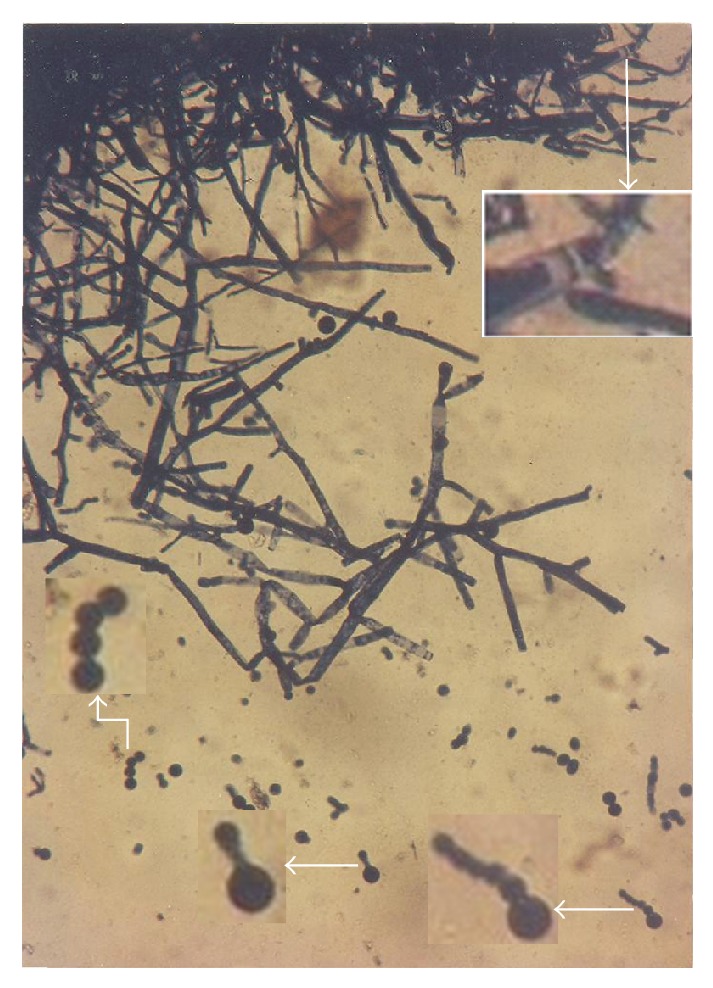
Different forms of thallic expression of *Mucor circinelloides *in glucose-substrated multiionic broth at ×200 mag. Inset: top right, thick septal wall; left, holoblastic conidia; bottom-left, growth sphere showing conidiogenous structure; bottom right, holothallic conidia.

A conspicuous feature in this study was the diversity of reproductive structures that arose from sporangiospores of *M. circinelloides* in submerged cultures. But more significant was the occurrence of septation in thallic structures. Morphological structures were recognized on the basis of delimiting septal formation or conidial ontogeny and conidial persistence. The nonpersistents remained as discrete units or, on budding by blastic action, soon released the daughter bud. Four types of persistent conidia were induced in the non-K^+^ incorporated media (control). These included (1) conidiogenous apparatus which consisted of numerous chains of globose and subglobose conidia concurrently originating from the outer wall of a double-walled vesicle, an apical enlargement of the conidiophore (Figure 5), similar to aspergillus head group but without the phialides, and here derived from olive-green and thick-walled septate hypha notched at the point of septation, Figure 6; (2) thallic conidia consisting of chains of rectangular to subglobose arthric formation after determinate thallic growth, Figures 7 and 8; (3) short or long chains of holothallic conidia arising from thallic conidiogenous structure, Figure 9; (4) holoblastic conidia, having blastic conidium ontogeny where all wall layers of the conidiogenous cell participatorily gave rise to short or long chains, Figure 9; individual globose/subglobose cells also abound the medium (Figure 9).

**Figure 10:**
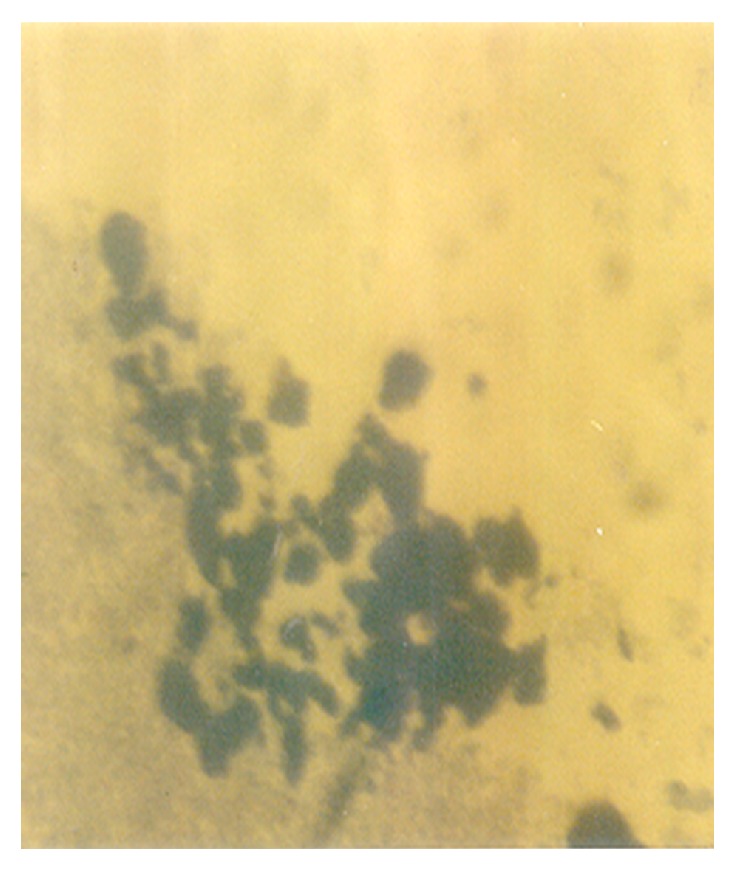
Clusters of cytoplasmic/granular/neoplastic units of *M. circinelloides *Tieghem induced in 0.50 g/L K^+^-mediated, glucose-substrate multiionic broth at ×1000 mag.

**Figure 11:**
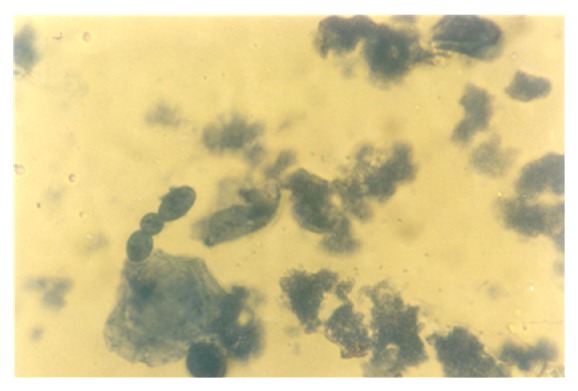
Holoblastic conidia and clusters of granular particles/neoplastic units of *M. circinelloides *Tieghem induced in 0.70 g/L K^+^-mediated, glucose-substrate multiionic broth at ×2000 mag.

**Figure 12:**
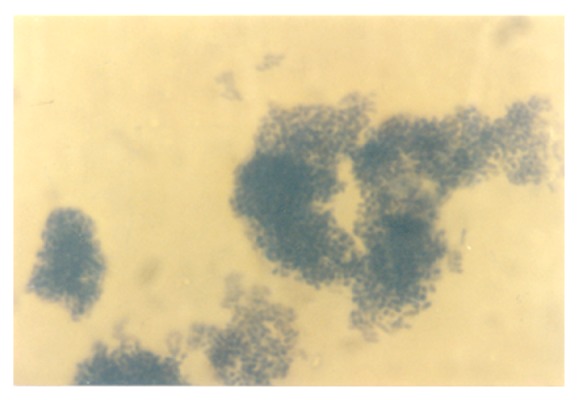
Globose and rod-shaped protoplasts of *M. circinelloides *Tieghem induced in 0.90 g/L K^+^-mediated, glucose-substrate multiionic broth at ×2000 mag.

 Although the aforementioned conidia were induced at 0.50 g/L K^+^ supplementation, they were more preponderant in the control experiments. In addition to these morphologies, granular units were numerous in the 0.5 g/L K^+^ supplemented medium (Figure 10). In the 0.7 g/L K^+^ medium, induced were holoblastic conidia, septate and double-walled thallic growth, thallic conidia, olive-green and septate thallic growth with conidiogenous vesicular head group, germlines, that is, growth sphere with short or extended germ tube, but more preponderant were granular units (Figure 11), which appeared larger in sizes in contrast to those observed in the 0.5 g/L K^+^ supplemented medium, and a few terminal budding yeast cells, which were present in varying shapes and sizes. Also present were septate and olive-green thallic growth with vesicular conidiogenous head group, holothallic, and holoblasic conidia; with further increase in K^+^ (0.9 g/L) supplementation, these conidia became fewer in number and there was a surfeit of granular unit-turned protoplasts as the units were now bounded by regular tender membrane but they could be globose or rod shaped (Figure 12). Also present were a few yeasts apparently just emerging, far less of which were terminal buddings. There was corresponding decrease in the occurrence of thallic conidia with increase in K^+^ level.

**Figure 13:**
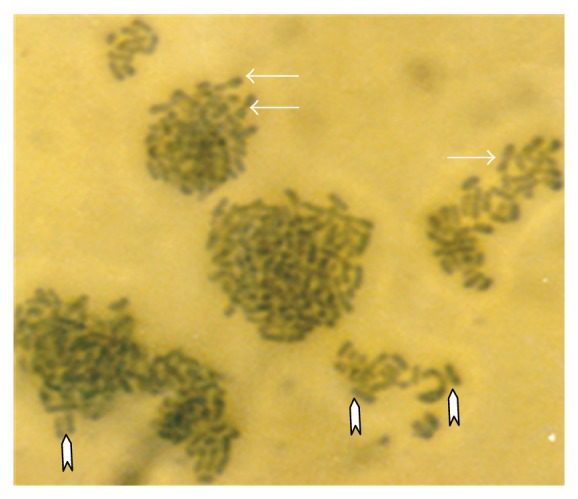
Protoplasts of *M. circinelloides *Tieghem induced in 1.00 g/L K^+^-mediated, glucose-substrate multiionic broth at ×2000 mag. Note the relative sizes compared to those of Figure 12; arrow, converting protoplast-to-nascent yeast; block arrow, cylindrical protoplasts.

**Figure 14:**
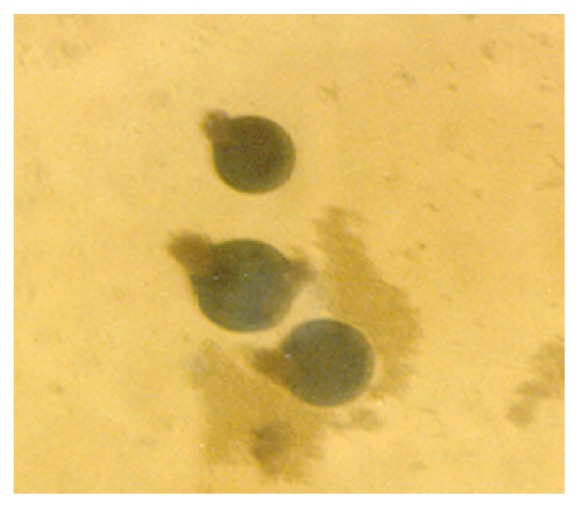
Terminal budding yeast cells of *M. circinelloides *Tieghem induced in 1.00 g/L K^+^-mediated, glucose-substrate multiionic broth at ×2000 mag.

**Figure 15:**
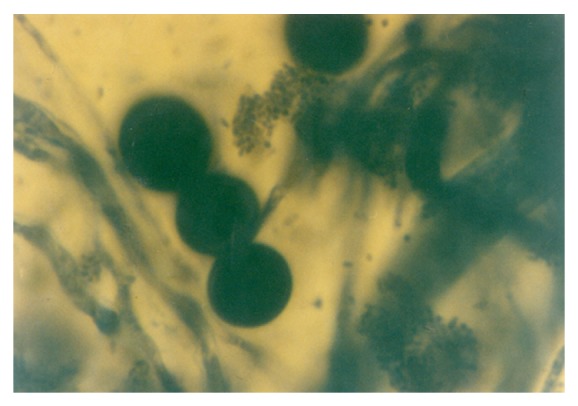
Protoplasts, holoblastic conidia and septate hyphae (out of focus) of *M. circinelloides* Tieghem coinduced in 1.00 g/L K^+^-mediated, glucose-substrate multiionic broth at ×1000 mag.

In the 1.0 g/L K^+^ supplemented medium, the protoplasts, now with conspicuous internal dimensions and singular binding membrane, had become larger in sizes (Figure 13). A few nascent/emergent yeast cells were observed, but preponderant were well-formed matured terminal budding yeast cells (Figure 14). Holoblastic conidia and septate and olive-green hyphae, with vesicular conidiogenous head group, were also present (Figure 15). 

**Figure 16:**
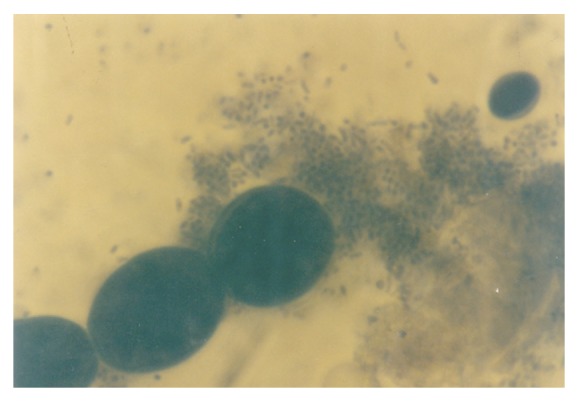
Protoplasts and holoblastic conidia of *M. circinelloides *Tieghem coinduced in 1.10 g/L  K^+^-mediated, glucose-substrated multiionic broth at ×2000 mag.

**Figure 17:**
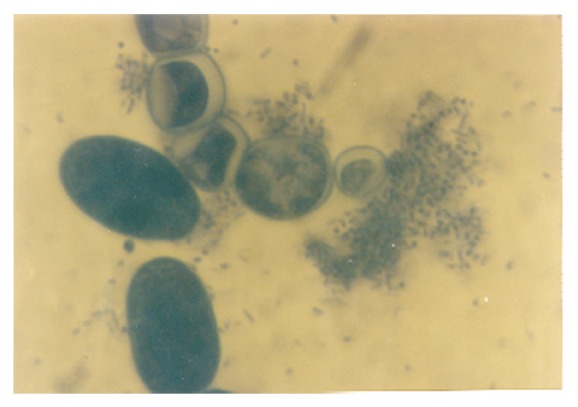
Protoplasts, holoblastic, and thallic conidia of *M. circinelloides *Tieghem coinduced in 1.10 g/L K^+^-mediated, glucose-substrated multiionic broth at ×2000 mag.

**Figure 18:**
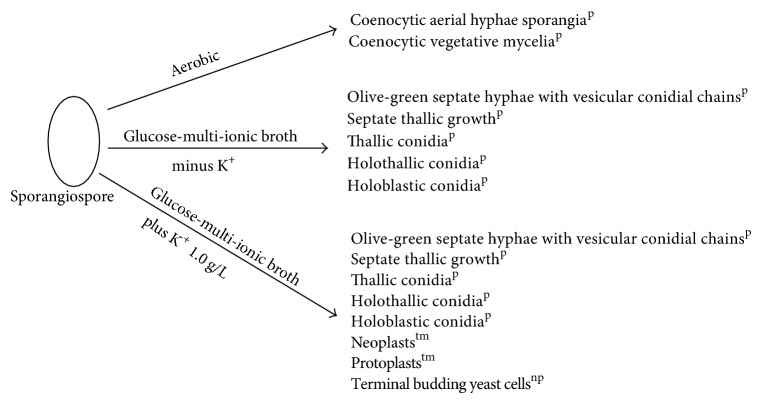
Scheme illustrating the morphologies induced from sporangiospores of *M. circinelloides *Tieghem, in this study; p: persistent conidia; tm: transient morphology; np: nonpersistent morphology

Conspicuous globose to short-rod protoplasts were also induced in the 1.10 gg/L K^+^ medium (Figure 16). They were however not as numerous as it was in the preceding level of K^+^concentration. The holoblastic conidia (Figures 16 and 17) and holothallic conidia (Figure 17) assumed more robust form at this level of K^+^ medium. Also present were germlines, double-walled thallic (septate) growth, septate and olive-green filament with vesicular conidiogenous apparatus, and nascent/emergent yeast cells, which appeared as rods and ellipsoids.  Figure 18 illustrated the various types of morphological forms of *M. circinelloides *as affected by K^+^ supplementation, observed in this study.

## 4. Discussion

That *M. circinelloides* is multifaceted has been proved with its conversion to multipolar budding yeast-like cells under CO_2_ pressure [4] or on induction with phenethyl alcohol [5], as well as thallic conidial production in Vogels medium [4, 5]. The latter developmental line is also expressed by other species of *Mucor, *as Bartnicki-Garcia and Nickerson [2] showed similar conidia ontogeny for *M. rouxii* (*M. indicus*), a morphological pattern exhibition said to be equivalent to ascomycetous Fungi imperfecti [12].

In the present study, *M. circinelloides* showed greater diversity of reproductive structures, representing different affinities of hyphomycetous pleomorphism. For beside thallic conidia, other structural forms induced in our synthetic broth include holothallic, holoblastic, septate mycelia conidiogenous apparatus with concurrent catenate conidia and terminal budding yeast cells, which is a characteristic of the Saccharomycetoideae. It is pertinent to note that yeast cells were not induced in our chemically defined medium unless K^+^ was incorporated.

A model for the induction of terminal budding yeast from sporangiospores of *M. circinelloides* has been presented [13]. The model details an assemblage of cryptic forms sequentially generated until the yeast morphology is attained. Biochemical changes lead to spore enlargement resulting in what is known as growth sphere [14]. The cytoplasm of growth spheres converts from apparent consistency to individualized, highly visible granular units. The occurrence of granular units in growth spheres has been previously recorded for *M. rouxii *[2]. Such spheroid with its cytoplasmic granular units, within a perhaps plastic wall, could be likened to tumours. This, perhaps, could be referred to as neoplasm. Adopting this coinage, neoplasm has also been observed in hyphal compartments and conidia in synthetic broth [6, 13, 15, 16]. When spheroidal wall lyse, hyphal wall rupture, or conidium bursts as a result of internal pressure, the neoplastic units released acquire individual life in the growth milieu [6, 13].

In the present study, neoplasts were not observed in the non-K^+^ medium but were generated and coexisted with divergent persistent asexual structures in the 0.5 g/L K^+^-broth. They clustered and slowly diffused as occurred at 0.7 g/L K^+^ concentration where observation showed relative increases in sizes. The conversion of individual neoplasts to protoplasts has been observed [6, 13, 15]. It was found to be a prelogarithmic growth event that occurred in an anisotropic growth environment [15, 16], which permitted intermedial ionic circulation [6]. Thus, conversion of growth spheres to neoplasts and subsequently protoplasts was enhanced at 1.0 g/L K^+^ : 0.1 g/L Na^+^ when the momentum of chemical potential became more favourable, in contrast to 0.9 g/L K^+^: 0.1 g/L Na^+^ or 1.10 g/L K^+^ : 0.1 g/L Na^+^[6]. 

In the present study, with further volume changes as occurred in the 0.9 g/L K^+^ broth, the units acquired internal dimensions, thus becoming globose or rod-shaped protoplasts. This coincided with the rise in magnitude of the H^+^ release intensity in contrast to the near—nil level recorded in the preceding K^+^-supplemented broth. But value of the H^+^ release intensity for the control broth was also maximal. What this indicated was that optimum and high level H^+^ release intensity as seen, respectively, in the control and 0.5 g/L K^+^ experiments did not cause the generation of neoplasts; instead, it was K^+^ that triggered it. This pervading biophysical presence has earlier been observed and attributed to K^+^ induction [6]. However, after neoplasm formation, increase in its (H^+^ release intensity) magnitude led to the conversion of neoplasts to protoplasts [6, 13, 16]. In the present study, this was further emphasized at 1.0 g/L K^+^ level, when the H^+^ release intensity was optimum and the protoplasts acquired more vigour, relative to that generated in the preceding 0.9 K^+^ broths.

Although persistent asexual structures recorded above were scantily present, neoplasts, protoplasts, and yeast cells were the predominant morphologies induced at 1.0 g/L K^+^ supplementation. Some nascent yeasts occurred as was also observed in the preceding K^+^ supplementation or at the 1.10 g/L K^+^-level, but the matured yeasts were more robust at 1.0 g/L K^+^ level and these became polar or bipolar budding.

That the bulk media initially tended toward alkalinity in K^+^ supplemented broths but subsequently decreased, becoming more acidic, possibly meant that there was initial influx of H^+^ and OH^−^ into the intracellular medium in order to mobilize metabolic activities in response to osmotic transport mechanisms. Perhaps, when this reached a state of neutrality in a carrier facilitated transport process, H^+^ were then extruded from the intracellular medium, against a concentration gradient, the bulk medium as in the present study, being buffered at acidic level. If we look at the 1.0 g/L K^+^-supplemented broth where protoplasts showed more vigour and yeast cells, more robustness but were also most preponderant in comparison with the other levels, the H^+^ release intensity profile assumed a two-phase construct. This has been previously reported [15]. That was in a study that involved physical counting of the induced yeast cells. The point of inflection in the pH profile also marked the lowest point (value) in the growth profile [15]. Significantly, that was the commencement of encounter with emergent yeast cells [15–17]. As seen in the present study, it also marked the inherent conversion of the evolved protoplasts into nascent yeasts. Thus, there was a congruence of H^+^ profile and progression leading to yeast form emergence, a process thought to be inherently stimulated at the point by the influx of H^+^ into the intracellular medium. It is significant to note that in a carbon substrate medium, this would necessarily entail H^+^-substrate symport [18, 19], an event that could be made possible through transmembrane pH gradient [15, 16]. 

That the H^+^ release intensity at point of inflexion in the pH profile in the control test was similar to that at 1.0 g/L K^+^-mediated medium indicated that similar transport mechanism occurred in both types of media. Yet, only thallic expression occurred in the control broth. This was not unexpected as C. L. Slayman and C. W. Slayman [20] showed that the filamentous microorganism, *Neurospora crassa,* permitted H^+^-substrate symport through its biomembrane, meaning the organism also permitted a transmembrane pH gradient in an anisotropic growth environment. But while neoplastic units, protoplasts, and yeast cells, except thallic growth, were completely absent in the control tests, they predominated in the 1.0 g/L K^+^-mediated broth. They were also present in the other K^+^-level mediated broths, although to a lesser extent. This further showed that K^+^ was of absolute necessity in the stimulation of formation of neoplasm, which subsequently led to protoplast generation in this transformative process, a confirmation of an earlier finding where K^+^ was found to be highly associated with Na^+^ during the morphogenetic transformation to terminal budding yeast [6]. 

In the phase 2 of the pH profile during the growth of *M. circinelloides*, the upward trend in the bulk medium pH was not sustained, except in the 1.0 g/L K^+^-mediated broth. The reason for this was not very clear. But the complex metabolic activities that could arise from the multiple morphologies in these media were perhaps attributable. Observation showed that with increase in K^+^ conc., there was consequent decrease in, as well as atrophying of, holoblastic conidia, holothallic conida, thallic conidia, and the olive-green and septate mycelia with vesicular conidial chains. However, the reverse was true at the highest level of K^+^ tested.

Since optical density measures the light absorbance capacity, the more thallic and blastic structures, (obviously larger in size and mass than neoplasts, protoplasts, and unicellular cells), that were in the medium, the greater the light absorbance value, which would consequently be reflected in the higher specific growth rate. The 1.10 g/L K^+^ medium had the highest growth rate while the 1.0 g/L K^+^ medium the least. This agreed with the experimental observation where the greater structural diversity, and robustness of persistent forms, occurred at 1.10 g/L K^+^, but neoplastic units, protoplasts, and nonpersistent yeast cells were the predominant morphologies induced at 1.0 g/L K^+^ incorporation. This level of K^+^ supplementation well nigh formed critical concentration with the broth composition of 0.10 g/L Na^+^, which permitted the maximum Na^+^ influx rate essential for exponential growth of induced yeast cells of *M. circinelloides *[6]. 

It has been argued that a transmembrane pH gradient is the platform for transport mechanisms during sporangiospore-yeast transformation [8, 9, 13, 15, 16]. In the present study, that there was H^+^ extrusion from the intracellular medium of the growing microorganism, *M. circinelloides*, also tended to anchor on the transport process being set up across the biomembrane since buffering at acidic level created slightly excess H^+^ on the outside of the membrane, and a deficit of H^+^ on the inner side [21–24]. It was within such buffered medium that initially facilitated diffusion and, subsequently, perhaps vectoral H^+^ extrusion, seen in the H^+^ release intensity, occurred. Magnitude of the H^+^ release intensity was optimal at 1.0 g/L and 0.0 g/L K^+^. But as has been pointed out, yeast cells did not occur in the latter broth.

**Figure 19:**
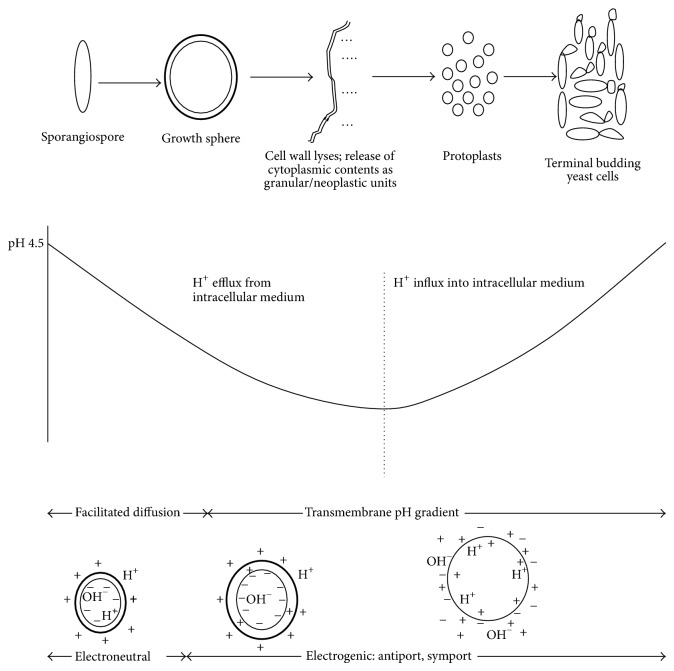
Scheme illustrating possible transport mechanisms during the growth of *M. circinelloides *Tieghem in buffered glucose-substrate multiionic broth for 120 h at pH 4.5, temp. 20°C, ambient.

Since K^+^ is exchanged for H^+^ [25–28], then ionic circulation was an inherent activity in this generative process; this has already been proved with K^+^-Na^+^ interaction, where at Na^+^ efflux there was simultaneous K^+^ influx at the prelogarithmic growth phase [6]. Therefore, the *M. circinelloides* spheroid and protoplast membranes were thought to be equipped with membrane-bound proton pumps which generated the electrochemical proton gradient across the membrane. The energy sequestered from this proton flow would be used to drive energy-requiring reactions. For instance, the enzyme ATP synthase could then synthesize ATP from ADP and inorganic phosphate. As in bacterial electrochemical proton gradient, the ATP generated could drive many transport processes, which would include the entry and exit of these ions [29]. Such harnessed energy could also be used for carrying out biosyntheses and lytic activities as well as other selected biosynthetic activities that occur intracellularly which could be triggered for directional physiology culminating in specific morphology. If we assume that this process occurred during the cultivation of *M. circinelloides*, then this was the Mitchellian proton pump [23]. It was thus greatly stimulated at 1.0 g/L K^+^ where there was a preponderance of protoplasts and terminal budding yeast cells.  Figure 19 illustrated the possible transport mechanisms that occurred during the transformation process of sporangiospores of *M. circinelloides* to yeast cells. 

Perhaps, part of the energy sequestered in this process was used for lytic activities on the spheroidal envelop by inherently induced lysozymes. After lyses of cell wall and the destruction of plasma membrane of growth spheres, granular cytoplasmic contents as neoplastic units were released into the medium. The orange-coloured granules did not take up the stain of trypan blue-in lactophenol, as has been previously observed [15–17], indicating that they were not protoplasmic in nature. That the units increased in sizes becoming coarse and at this stage took up the stain, and subsequently assuming a regular form, globose, subglobose, or short rods, indicated that they had become protoplasmic [13, 15–17]. The increase in the bulk medium pH at pH-profile phase 2 suggested that intermedial ionic communication [6] and hence chemiosmotic processes as occurs in all biomembranes [30] continued with H^+^ influx into the intracellular medium. Hence, with full complement of the proton motive force [24], this ensured H^+^ substrate symport as has been demonstrated for *Escherichia coli *[18, 19] and* Metschnikowia reukaufii* [31] which, respectively, transport lactose and glucose into the intracellular medium in a 1 : 1 stoichiometric relationship. 

Since glucose was the only preformed organic source incorporated into the broth used in the cultivation of *M. circinelloides *in this study, then it was transported into the intracellular medium of the protoplasts, beginning early or from inflexion point, then onward in the second phase of the pH profile where it could be utilized for sundry biochemical activities, including the fundamental construction of cytoskeletal structures, thus leading to cell wall biogenesis, hence impacting the yeast form and, subsequently, asexual reproduction, that is, daughter bud formation. A possibility for exponential growth, as occurs with *Saccharomyces cerevisiae *describing sigmoid pattern, could thereafter ensue. The description of sigmoid growth pattern by induced terminal budding yeasts of *M. circinelloides* has been demonstrated in several studies. But in such studies other incorporated medial components became critical, including myoinositol and uracil [6, 8, 9].

## 5. Conclusion 

This paper shows that pleomorphism in *M. circinelloides* is multidirectional. It further demonstrates the possibility of inducing terminal budding yeast cells from filamentous microorganisms. It represents a deviant method of yeast ontogeny. It has already been demonstrated for another zygomycete, which is *Rhizopus stolonifer, *in a study in which Ca^2+^ was found to play significant role in terminal budding yeast expression [32, 33].
